# Cyst of the canal of Nuck in an adult female patient: A case report on surgical management

**DOI:** 10.1016/j.ijscr.2024.110807

**Published:** 2024-12-31

**Authors:** Caterina Maria Nava, Benoit Geng, Alexis Litchinko, Claudia Jaccard, Bernhard Egger

**Affiliations:** Department of Surgery, HFR Fribourg Cantonal Hospital, CH-1708 Fribourg, Switzerland; Department of Pathology, Promed Laboratoire Médical SA, CH-1723 Marly, Switzerland

**Keywords:** Cyst of the canal of Nuck, Female hydrocele, Inguinal mass, Case report

## Abstract

**Introduction:**

The cyst of the canal of Nuck is a rare cause of inguino-labial swelling in adult women, arising from an obliteration failure of the processus vaginalis during embryological development. Its rarity often leads to misdiagnosis and improper treatment. This article highlights its diagnosis and surgical management.

**Case presentation:**

A 21-year-old woman presented with a two-month history of symptomatic swelling in the right inguino-labial region, after consulting multiple doctors. Various investigations including abdominal ultrasonography, computed tomography, and Magnetic Resonance Imaging revealed multiloculated cystic lesion with thin walls, without communication to the peritoneal cavity. During surgery, an encysted sack was discovered at the external inguinal ring, with no associated hernia. The cyst was ligated high and excised along with the round ligament. The anatomical defect was repaired without using prosthetic mesh. The patient had an uneventful post-operative recovery at six-months follow-up.

**Discussion:**

The encysted hydrocele (Type 1) is the most common among the three types of canal of Nuck hydroceles. This rare entity should be considered in the differential diagnosis of groin masses, even in adult women. Ultrasonography is often used initially, while Magnetic Resonance Imaging is reserved for complex cases. Surgical intervention is essential for both diagnosis and treatment, with dissection extending up to the deep inguinal ring to address any associated hernias. The choice between open or laparoscopic procedures depends on the nature of the defect.

**Conclusion:**

Surgery remains the sole standard therapeutic approach for the management of the cyst of the canal of Nuck.

## Highlights

• Nuck canal cysts are rare but must be considered in adult female inguinal masses.
• Diagnosis requires imaging differentiation from hernias and other lesions.
• Definitive treatment is surgical, differing by Nuck cyst type.
• Open approach suits Type I, laparoscopic for Type II, and tailored for Type III.
• In anterior approach, inspect for hernias, repair defects, and preserve nerves.

## 1 Introduction

The canal of Nuck is an anatomical space described in females as the analogue of the processus vaginalis in males, an evagination of the parietal peritoneum that traverses the inguinal canal along with the round ligament of the uterus into the labia majora [1]. The processus vaginalis normally completely obliterates during the first five years of life. Thus, abnormalities of the canal of Nuck are mostly encountered in young patients and are rarely present in adults [2].

The aim of this case report is to highlight the diagnosis and surgical technique for treating cysts of the canal of Nuck in adult patients. The clinical importance of differentiating Nuck cysts from other inguinal swellings is crucial to prevent unnecessary investigations and avoid inappropriate surgical interventions, such as mesh placement.

Herein, we present a rare occurrence of a Nuck cyst in an adult woman, diagnosed at a community hospital during a dedicated surgical outpatient consultation. This work is reported in line with the SCARE criteria [3].

## 2 Case presentation

A 21-year-old healthy woman presented to our surgical clinic with a 2-month history of persistently painful and palpable swelling in the right inguinolabial region, which worsened in standing position. This discomfort led to multiple consultations with her family doctor, gynecologist, and three visits to the emergency department within one month. After various radiological investigations, she was referred to our surgical outpatient clinic.

The patient did not report any associated abdominal pain or nausea. She had no history of previous surgeries, no diagnosis of endometriosis or other gynecological conditions, and her menstrual cycles were regular.

On physical examination, a well-defined, firm, oval swelling was noted on the inner aspect of the anterosuperior iliac spine. The mass was irreducible, with the skin and subcutaneous tissue freely mobile over it. The swelling did not increase in size during Valsalva maneuvers, and no hernia was detected on examination. All other examination findings were unremarkable.

**Fig. 1:**
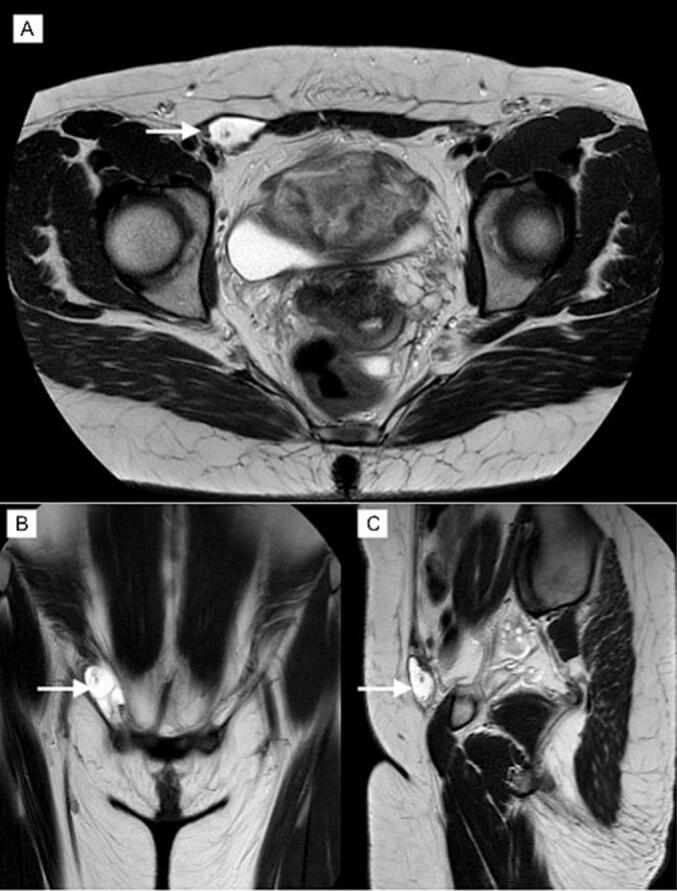
T2-weighted MRI in transverse (A), coronal (B), and sagittal (C). An arrow indicates a 4 × 2.6 × 1.7 cm cystic lesion with thin septations and no communication to the peritoneal cavity.

Magnetic Resonance Imaging and abdominal ultrasound revealed a well-defined cystic structure with multiple septations and thin walls, measuring 4 × 2.6 × 1.7 cm. It was located in the lower part of the right oblique muscle, with no apparent connection to the peritoneal cavity, and extended into the right inguinal canal. The structure displayed a liquid signal, no diffusion restriction, and no abnormal contrast enhancement after intravenous gadolinium administration (Fig. 1).

A differential diagnosis of an inguinal hernia or hydrocele of the canal of Nuck was made, and the patient was scheduled for elective surgery.

The surgery was performed under general anesthesia. The groin was explored starting with a transverse skin-crease incision for an anterior approach. After identification of the external inguinal ring, and the incision of the external oblique muscle's fascia, the cyst was clearly visible as a 7 cm-long and 2 cm-wide encysted sack extending to the labia majora, parallel to the round ligament, with partial obliteration of the canal of Nuck at the midpoint. This lesion had no communication with the abdominal cavity, did not cause distension of the deep inguinal ring or transversalis fascia and no associated inguinal hernia was found. The cyst was carefully dissected from the ilio-inguinal nerve, which was potentially the source of the patient's pain, and the nerve was preserved. An *in toto* resection of the canal of Nuck required the ligation of both the canal and the round ligament near the deep inguinal ring. The anatomical defect was repaired without the use of prosthetic mesh, and the wound was closed in layers.

The postoperative period was uneventful, and at the six-month follow-up, there was no recurrence of the mass or pain.

**Fig. 2:**
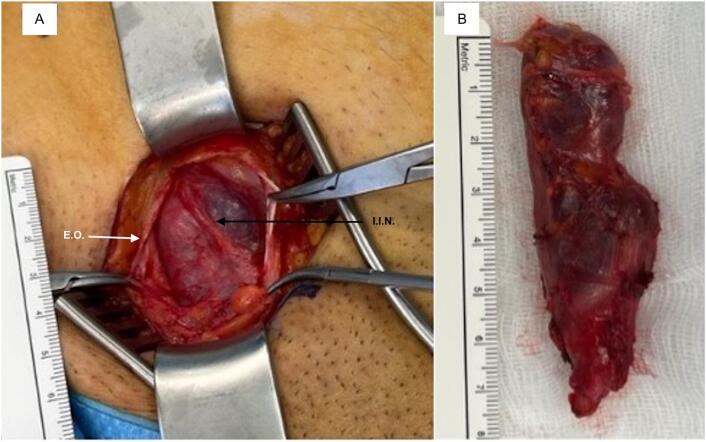
(A) Intraoperative view of the cyst and ilioinguinal nerve (I.I.N) after incision of the external oblique (E.O.) muscle's fascia (B) Resected canal of Nuck cyst showing brown fluid content after incision. (For interpretation of the references to colour in this figure legend, the reader is referred to the web version of this article.)

Macroscopically, the excised tissue resembled a saccular fragment of 7.2 cm in length and between 0.9 cm and 2.6 cm in width, with a smooth whiteish surface covered by a delicate vascular network and adipose tissue. The cut surface showed a cyst with brown liquid content (Fig. 2).

**Fig. 3:**
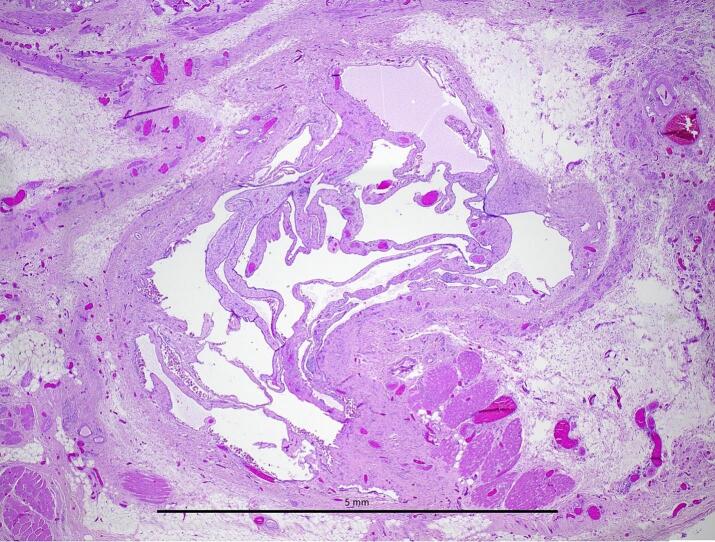
Hematoxylin and eosin-stained histological section of the excised cyst, with flat and rarely cuboidal mesothelial lining, consistent with hydrocele cyst of the canal of Nuck.

Histologically, there was a multi-loculated cyst with septations containing small blood vessels. The septations were covered by flat and rarely cuboidal cells and surrounded by an infiltrate composed of neutrophils, lymphocytes, plasma cells, histiocytes, hemosiderin and fibrolipomatous connective tissue. Immunohistochemically, the lining cells were positive for WT1 and negative for estrogen receptor. No stromal spindle cells were identified and immunohistochemistry for CD10 showed a nonspecific pattern highlighting myofibroblasts (Fig. 3).

## 3 Discussion

The processus vaginalis is a pouch-like extension of the parietal peritoneum into the inguinal canal, that facilitates the descent of ovaries during fetal development [1,4,5]. Usually, the superior part of this outpouch obliterates in a craniocaudal axis before the first year of life. In rare cases, this obliteration fails completely or partially, leading to a persistent canal of Nuck. In such cases, mesothelial cells lining the canal secrete fluid that accumulates within this space [6].

The canal of Nuck was first described in 1691 by the Dutch anatomist Anton Nuck [7]. The most commonly used classification, outlined by Counseller and Black categorizes the canal of Nuck into three types: encysted, communicating, and bilocular hydroceles [1,8,9].

The encysted hydrocele of the canal of Nuck (Type 1) is the most common variant. It does not communicate with the peritoneal cavity, and results from the partial obliteration of only the proximal portion of the canal of Nuck. It can be located anywhere along the round ligament, from the internal ring to the vulva. Clinically, it presents as a non-reducible, painless mass whose volume does not change with the Valsalva maneuver [10].

The communicating hydrocele (Type 2) closely resembles to the congenital hydrocele in males, with an open communication to the peritoneal cavity, which carries the potential for herniation. It presents as a reducible mass that may appear only after performing the Valsalva maneuver or upon standing [11].

The bilocular hydrocele (Type 3), the least common variant, occurs when there is an encysted inferior section in the inguinal canal and labia majora, as well as an upper intraabdominal portion. The hydrocele is compressed by the deep inguinal ring, creating an hourglass appearance [1].

Our case belonged to a Type 1 according to the Counseller and Black classification.

A summary of four recent studies on Nuck cysts' management is summarized in Table 1 [[12], [13], [14], [15]]. Approaches vary based on cyst type and surgical expertise. While use of a prosthetic mesh remains debated, some authors advocate routine mesh reinforcement to reduce postoperative hernias, given the internal ring widening caused by the cyst [12,16].

**Table 1:** Summary: Management of cysts of the canal of Nuck.

Author	Year of publication	Type of publication	Type of cyst	Procedure	Hernia associated	Mesh use
Venkateswaran et al. [12]	2024	Retrospective study (20 patients)	15 Type I 5 Type III	10 TAPP 10 open repair: high ligation	11/20 Yes	20/20 Yes
Gkioulos et al. [14]	2023	Single case report	Type I	Open repair High ligation	No	No
Aldhafeeri et al. [15]	2023	Single case report	Type III	Open repair High ligation	No	Yes
Matsumoto et al. [13]	2014	Single case report	NS	TEP	Yes	Yes

TAPP: transabdominal pre-peritoneal, TEP: totally extraperitoneal, NS: not specified.

Typically, the cyst of the canal of Nuck presents as a painless or moderately painful (when tense or infected), translucent, and nonreducible lump in the inguino-labial region, without associated nausea or vomiting [17]. Symptoms may be chronic or acute. In women, differential diagnoses for inguinal swelling include indirect inguinal hernia, lymphadenopathy, Bartholin's cyst, infection or abscess, endometriosis of the round ligament, and tumors (lipoma, leiomyoma, and sarcoma) [4,11,18].

Due to its cost-effectiveness and widespread availability, ultrasonography is often employed for initial imaging assessments, while Magnetic Resonance Imaging is reserved for more complex cases and further investigation and shows a cystic lesion hyperintense on T2 and hypointense on T1 [11,18]. In cases of hydroceles, sonographic findings typically reveal an elongated mass containing anechoic fluid [19]. On occasion, a Valsalva maneuver may alter the contents of a hernia, while an encysted hydrocele remains unaffected [20].

The management of a cyst of the canal of Nuck necessitates surgical intervention, which is essential for both diagnosis and treatment [1]. Surgical repair encompasses the exploration of the inguinal canal, the resection and proximal ligation of the hydrocele, and the correction of any parietal peritoneal defects. The choice between open or laparoscopic procedures is contingent on the nature of the defect: external Type I Nuck's hydroceles should be treated using an open surgical technique, while a laparoscopic approach (totally extraperitoneal-TEP- or transabdominal pre-peritoneal- TAPP) is more suitable for intra-abdominal Type 2 Nuck hydroceles [13]. Complex cases, such as Type 3, require individual assessment due to their difficulty, with surgical success largely depending on the surgeon's expertise [13,17]. Given the common association of hydrocele with indirect inguinal hernias, dissection should extend up to the deep inguinal ring, accompanied by the high ligation of the peritoneal pouch neck [1].

This case report details a Type I hydrocele operation emphasizing meticulous nerve preservation and avoiding mesh placement when unnecessary. It further enriches the limited literature available on this rare entity.

## 4 Conclusion

In conclusion, the cyst of the canal of Nuck is seldom thoroughly explored in surgical and gynecological literature, and it remains a relatively unfamiliar condition among medical practitioners. This case highlights the importance of considering it as a differential diagnosis when evaluating inguinal swelling in female adults, while also detailing the surgical approach.

Ultrasonography is a valuable first-line imaging tool to distinguish the hydrocele of the canal of Nuck from other conditions and can be supplemented with magnetic resonance imaging when available.

Definitive diagnosis and treatment typically involve *in toto* hydrocelectomy, with hernioplasty required if an inguinal hernia is also present. The decision between an open or laparoscopic procedure depends on the nature of the defect.

## Author contribution

The first three authors contributed to the writing, design, data collection, reviewing and editing of this paper. The fourth author contributed to the writing of the pathology segment.

The last author contributed to the design, supervision and validation of this paper. All authors approved the final manuscript.

## Informed consent

Written informed consent was obtained from the patient for publication of this case report and accompanying images. On request, a copy of the written consent is available for reviewers of this journal.

## Ethical approval

The competent ethics committee of our institution does not require ethical approval for reporting individual cases.

## Guarantor

The guarantor for this statement is Dr. Caterina Maria Nava.

## Research registration number

This study is not a “First in Man” study.

## Provenance and peer review

Not commissioned, externally peer reviewed.

## Funding

This research did not receive any specific grant from funding agencies in the public, commercial, or not-for-profit sectors.

## Conflict of interest statement

All authors declare no conflict of interest.
